# Structural dynamics of CH_3_NH_3_^+^ and PbBr_3_^−^ in tetragonal and cubic phases of CH_3_NH_3_PbBr_3_ hybrid perovskite by nuclear magnetic resonance

**DOI:** 10.1038/s41598-020-70128-5

**Published:** 2020-08-04

**Authors:** Ae Ran Lim, Sun Ha Kim, Yong Lak Joo

**Affiliations:** 1 Analytical Laboratory of Advanced Ferroelectric Crystals, Department of Science Education, Jeonju University, Jeonju, 55069 Korea; 2 Seoul Western Center, Korea Basic Science Institute, Seoul, 03759 Korea; 3 Department of Chemistry, Kyungpook National University, Daegu, 41566 Korea; 4 Robert Fredrick Smith School of Chemical and Biomolecular Engineering, Cornell University, Ithaca, NY 14853 USA

**Keywords:** Materials science, Physics

## Abstract

Understanding the structural dynamics of lead-halide perovskites is essential for their advanced use as photovoltaics. Here, the structural dynamics of the CH_3_NH_3_ cation and PbBr_6_ octahedra in the perovskite CH_3_NH_3_PbBr_3_ were studied via nuclear magnetic resonance (NMR) to determine the mechanism of the transition from the tetragonal to cubic phase. The chemical shifts were obtained by ^1^H, ^13^C, and ^207^Pb magic angle spinning NMR and ^14^N static NMR. The chemical shifts of the ^1^H nuclei in CH_3_ and NH_3_ remained constant with increasing temperature, whereas those of the ^13^C and ^207^Pb nuclei varied near the phase transition temperature (*T*_C_ = 236 K), indicating that the structural environments of ^13^C and ^207^Pb change near *T*_C_. The spin–lattice relaxation time T_1ρ_ values for ^1^H, ^13^C, and ^207^Pb nuclei increased with increasing temperature and did not exhibit an abrupt change near *T*_C_. In addition, the two lines in the ^14^N NMR spectra superposed into one line near *T*_C_, indicating the occurrence of a phase transition to a cubic phase with higher symmetry than tetragonal. Consequently, the main factor causing the phase transition from the tetragonal to cubic phase near *T*_C_ is a change in the surroundings of the ^207^Pb nuclei in the PbBr_6_ octahedra and of the C–N groups in the CH_3_NH_3_ cations.

## Introduction

**Figure 1:**
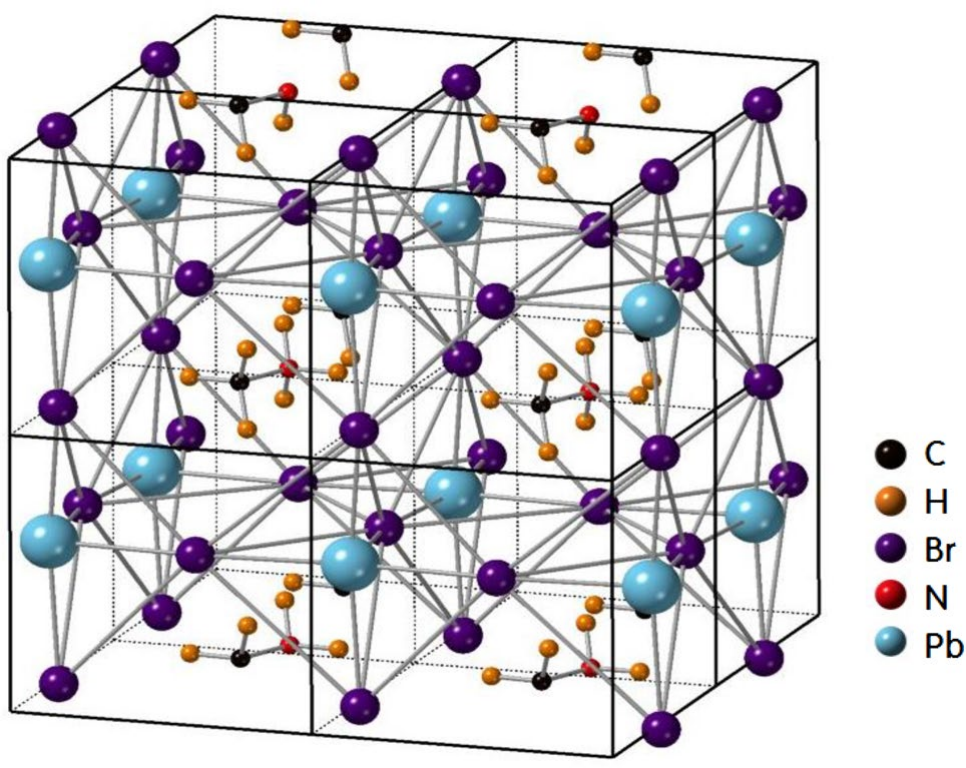
Crystal structure of cubic phase CH_3_NH_3_PbBr_3_. The software used to create the Fig. is CrystalMaker Software.

Lead-halide perovskites currently represent the most promising photovoltaic materials for the production of low-cost, high-performance solar cells^1,2^. In recent years, researchers have succeeded in significantly improving the power conversion efficiency (PCE) of this hybrid perovskite, and rapid advances in this field led to a record-high PCE. The very important for optoelectronic heterostructures are solar cells, photodetectors, and laser diodes^3–6^. For this class of materials, which has the general formula CH_3_NH_3_Pb*X*_3_ (*X* = Cl, Br, and I), an inorganic cage of Pb*X*_6_ octahedra encloses an organic cation at the CH_3_NH_3_^+^ site^7–12^. The phase transition temperatures of CH_3_NH_3_PbBr_3_, a representative perovskite, are 148.8, 154, and 236.3 K, corresponding to a total of four crystal phases^13,14^; with decreasing temperature, the cubic phase (I) transforms to a tetragonal phase (II) at 236.3 K, to another tetragonal phase (III) at 154 K, and finally to an orthorhombic phase (IV) at 148.8 K. In tetragonal phase II, the CH_3_NH_3_^+^ ions undergo isotropic reorientation, whereas in the lower-temperature phases, the reorientation of C–N axes seems to be frozen^15^. All the phase transitions are first-order and order–disorder type, although the highest temperature transition is close to second-order. From the high-temperature cubic phase with freely rotating CH_3_NH_3_ cations, this compound enters lower-symmetry tetragonal phases and finally a low-temperature orthorhombic phase with the orientation of CH_3_NH_3_ cations fixed at ordered positions^16–20^. At room temperature, the structure is cubic, the space group is Pm3m, and the lattice constant *a* = 5.93129 Å and *Z* = 1^13^. In this crystal structure, there exists a CH_3_NH_3_^+^ cation at the centre of a cube formed by corner-sharing PbBr_6_ octahedra^18,21^, as shown in Fig. 1. Below 236 K, the crystal structure has a tetragonal and belongs to the space group I4/mcm with lattice constants *a* = *b* = 8.32 Å, *c* = 11.83 Å, and *Z* = 4^22^. Throughout the transition from the tetragonal II phase to the tetragonal III phase at 154 K, the full width at half maximum of the Raman ν_6_ band shows an abrupt increase^8^. At lower temperatures, CH_3_NH_3_PbBr_3_ undergoes a first-order structural phase transition from the tetragonal III (I4/mmm) phase to the orthorhombic IV (Pnma) phase^9^.

In a previous nuclear magnetic resonance (NMR) investigation, the temperature dependence of ^81^Br nuclear quadrupole resonance frequencies and ^1^H spin–lattice relaxation times in the laboratory frame T_1_ for CH_3_NH_3_PbBr_3_ were discussed by Xu et al.^23^ According to their results, the two ^81^Br NQR lines in phase II were reduced to one line in phase I. The discontinuity of the NQR line at this transition point implied a first-order transition. ^1^H T_1_ varied continuously, and no discernible change in the free induction decay was observed during the I–II transition. The phase transition had no significant effect on the motional state of the CH_3_NH_3_^+^ ions. Furthermore, Baikie et al.^13^ reported that the ^1^H magic angle spinning (MAS) NMR spectra showed two clear peaks corresponding to the CH_3_ and NH_3_ environments in the high-temperature phase, and the ^1^H and ^13^C NMR spectra of CH_3_NH_3_PbBr_3_ showed that the CH_3_NH_3_^+^ units undergo dynamic reorientation.

Measuring the spin–lattice relaxation time in the rotating frame T_1ρ_ by MAS NMR allows for the probing of molecular motion in the kHz range, whereas the spin–lattice relaxation time in the laboratory frame T_1_ measured by static NMR reflects motion in the MHz range. Although the ^1^H T_1_ of CH_3_NH_3_PbBr_3_ has been examined by a few research groups, the corresponding phenomena by ^1^H, ^13^C, and ^207^Pb MAS NMR spectra and T_1ρ_ have not been fully studied. In addition, information regarding ^14^N in the CH_3_NH_3_ cation has not yet been discussed.

In the present study, the structural dynamics of the CH_3_NH_3_ cation and PbBr_6_ octahedra in CH_3_NH_3_PbBr_3_ were studied in detail by NMR to resolve the phase transition mechanisms from the tetragonal phase to the higher-temperature cubic phase. The temperature dependences of the chemical shifts and spin–lattice relaxation time in the rotating frame T_1ρ_ were measured using ^1^H MAS NMR, ^13^C cross-polarization (CP)/MAS NMR, and ^207^Pb MAS NMR with emphasis on the role of the CH_3_NH_3_ cation and PbBr_6_ octahedra in CH_3_NH_3_PbBr_3_. In addition, the ^14^N static NMR spectra of CH_3_NH_3_PbBr_3_ in the laboratory frame were acquired near the phase transition temperature. The abovementioned results help in understanding the thermal stability and the structural dynamics based on the phase transition mechanism, towards the practical application of this material.

## Experimental

CH_3_NH_3_Br and PbBr_2_ were dissolved in a dimethylformamide solution and heated the mixed suspension on a hot plate to obtain a transparent solution. Detailed methods for the crystal growth are given elsewhere^21,24,25^. The CH_3_NH_3_PbBr_3_ single crystals obtained here were orange in colour with a square shape.

Differential scanning calorimetry (DSC) (TA, DSC 25) was conducted at a heating rate of 10 °C/min over a temperature range from 190 to 525 K under nitrogen gas. Thermogravimetric analysis (TGA) was performed on a thermogravimetric analyser (TA Instrument) in an interval from 300 to 780 K at a heating rate of 10 °C/min. Approximately 11.15 mg of CH_3_NH_3_PbBr_3_ was used in each experiment.

NMR measurements were carried out at 9.4 T using a Bruker 400 MHz Avance II + spectrometer at the Korea Basic Science Institute, Western Seoul Center. The ^1^H, ^13^C, and ^207^Pb NMR frequencies were 400.13, 100.61, and 83.75 MHz, respectively. Powdered samples were packed in zirconia MAS rotors with Macor caps, and the MAS rate was set to 10 kHz for the ^1^H MAS, ^13^C MAS, and ^207^Pb MAS NMR measurements to minimise spinning sideband overlap. The spin–lattice relaxation time in the rotating frame T_1ρ_ was measured using an inversion recovery pulse sequence, which employs compensating pulses. The ^13^C T_1ρ_ values were measured by varying the duration of the ^13^C spin-locking pulse applied after the CP preparation period. The width of the π/2 pulse used for measuring T_1ρ_ of ^1^H and ^13^C was 3.45 µs, and that for measuring T_1ρ_ of ^207^Pb was 3.5 µs. In addition, ^14^N NMR spectra of a CH_3_NH_3_PbBr_3_ single crystal were measured with a Larmor frequency of 28.90 MHz in the laboratory frame.

Temperature-dependent NMR spectra were recorded over 180 to 430 K; the NMR spectra and relaxation times could not be measured outside this temperature range because of the limitations of the spectrometer. Sample temperatures were held constant within ± 0.5 K by controlling the nitrogen gas flow and heating current.

## Experimental results

**Figure 2:**
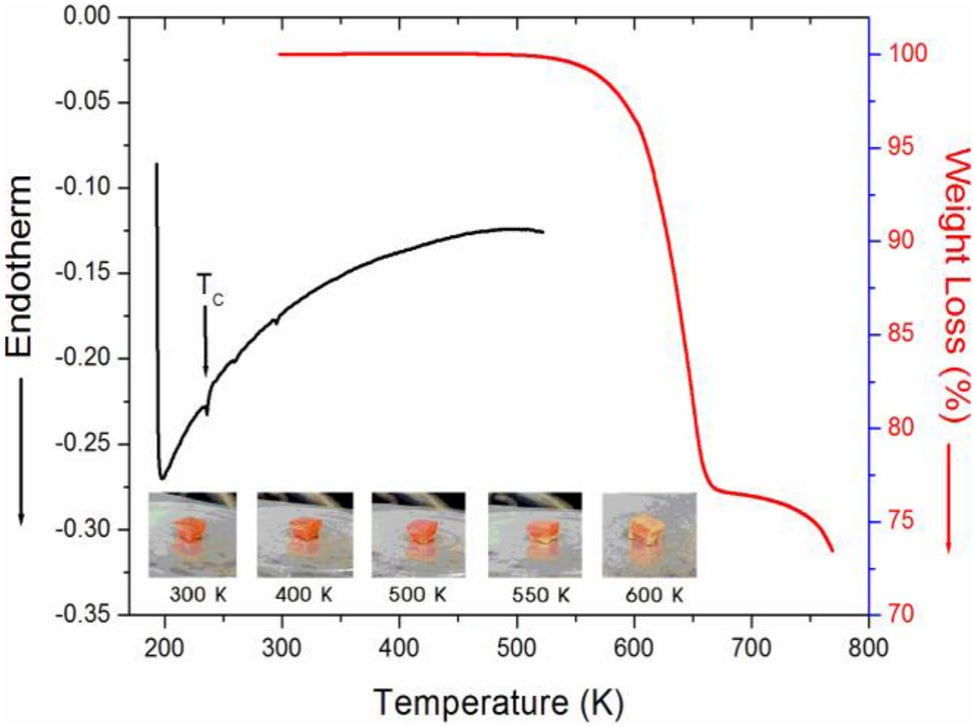
Differential scanning calorimetry (DSC) and thermogravi-metric analysis (TGA) of CH_3_NH_3_PbBr_3_ (inset: color changes of CH_3_NH_3_PbBr_3_ single crystal according to the temperature).

Figure 2 shows the DSC and TGA curves obtained under a nitrogen atmosphere. DSC analysis was used to determine the phase transition temperature; only one endothermic peak related to a phase transition was observed at 236 K, which is consistent with previously reported *T*_C_ values.^13,14^ The thermal stability of CH_3_NH_3_PbBr_3_ was examined by TGA. The first occurrence of mass loss began at approximately 530 K, which represents the onset of partial thermal decomposition. The mass sharply decreased between 550 and 650 K, with a corresponding mass loss of 22% near 650 K. Optical polarizing microscopy experiments were also conducted to further understand the thermal stability at high temperatures. The colour of the crystal was orange at room temperature, as shown in the inset in Fig. 2. As the temperature increased, the state of the crystal remained the same from 400 to 500 K. Above 550 K, a slight opacity occurred at the bottom of the crystal, and at approximately 600 K, the crystal was nearly opaque.

**Figure 3:**
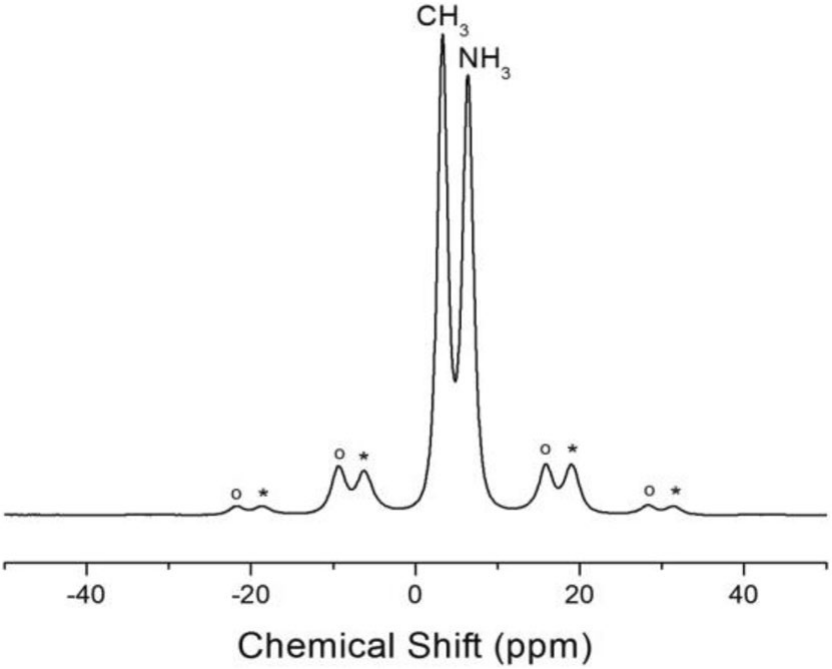
^1^H MAS NMR spectrum for CH_3_ and NH_3_ of CH_3_NH_3_PbBr_3_ at 300 K, and the spinning sidebands are marked with open circles and asterisks.

The ^1^H NMR spectrum of CH_3_NH_3_PbBr_3_ was recorded by MAS NMR at a frequency of 400.13 MHz. Figure 3 shows the ^1^H MAS NMR spectrum at 300 K, where the spinning sidebands are marked with open circles and asterisks. The two peaks in the ^1^H spectrum correspond to CH_3_ and NH_3_ environments, with the chemical shifts at δ = 3.27 and 6.36 ppm assigned to ^1^H in CH_3_ and NH_3_, respectively. The chemical shifts remained quasi-constant with increasing temperature, indicating that the structural environments of ^1^H in the CH_3_ and NH_3_ groups were unchanged (see the Supplementary Information). Additionally, the line width (full-width at half-maximum) of the ^1^H MAS NMR signal at 300 K is approximately 1.62 ppm, which also remained nearly constant with temperature change.

**Figure 4:**
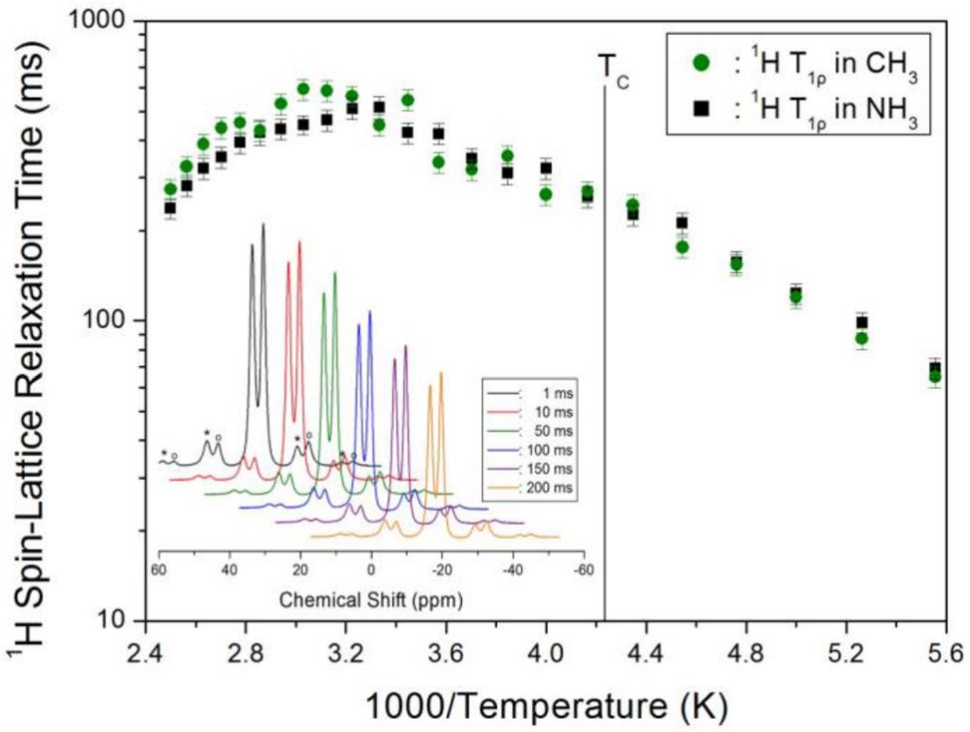
^1^H spin–lattice relaxation times T_1ρ_ for the CH_3_ and NH_3_ ions of CH_3_NH_3_PbBr_3_ as a function of inverse temperature (inset: recovery plots of the ^1^H MAS NMR spectrum by delay time at 300 K).

The ^1^H inversion-recovery curves for both CH_3_ and NH_3_ at each temperature were fitted to exponentials to extract T_1ρ_. The data were well fitted a single exponential, indicating that there is one dominant relaxation mechanism acting per environment. Thus, T_1ρ_ was determined by fitting the decay plots with the equation below^26,27^.

1 $${\text{P}}(t)/{\text{P}}_{0} = \exp \left( { - t/{\text{T}}_{{1\rho }} } \right),$$

where P(*t*) is the magnetisation, *t* is the spin-locking pulse duration, and P_0_ is the total nuclear magnetisation of ^1^H at thermal equilibrium. The recovery curves of ^1^H in CH_3_NH_3_PbBr_3_ were measured for various delay times at each temperature. Figure 4 (inset) shows the recovery traces for ^1^H measured for delay times ranging from 1 to 200 ms at 300 K. The intensity of the recovery traces differed with delay time. The T_1ρ_ values obtained from the intensity versus delay time and shown in Fig. 4 reveal that T_1ρ_ increased with temperature because proton hopping was accelerated. This is in agreement with Xu et al.^23^, who reported that the ^1^H T_1_ increased smoothly with increasing temperature through the high-temperature phase transition. The T_1ρ_ values of ^1^H in CH_3_ and NH_3_ in the CH_3_NH_3_^+^ cation show similar trends with temperature and are nearly the same within the error range. The T_1ρ_ values show no change near the phase transition temperature (*T*_C_ = 236 K). T_1ρ_ increased with increasing temperature, reaching the maximum values of 592 ms and 456 ms for CH_3_ and NH_3_, respectively, near 330 K above the phase transition temperature, and then decreased with increasing temperature. Although the structural environment of ^1^H in the CH_3_NH_3_ groups does not change with temperature, their molecular motion increases at high temperatures, as indicated by the T_1ρ_ values. Above *T*_*C*_, the ^1^H T_1ρ_ value for CH_3_ slightly exceed that for NH_3_.

**Figure 5:**
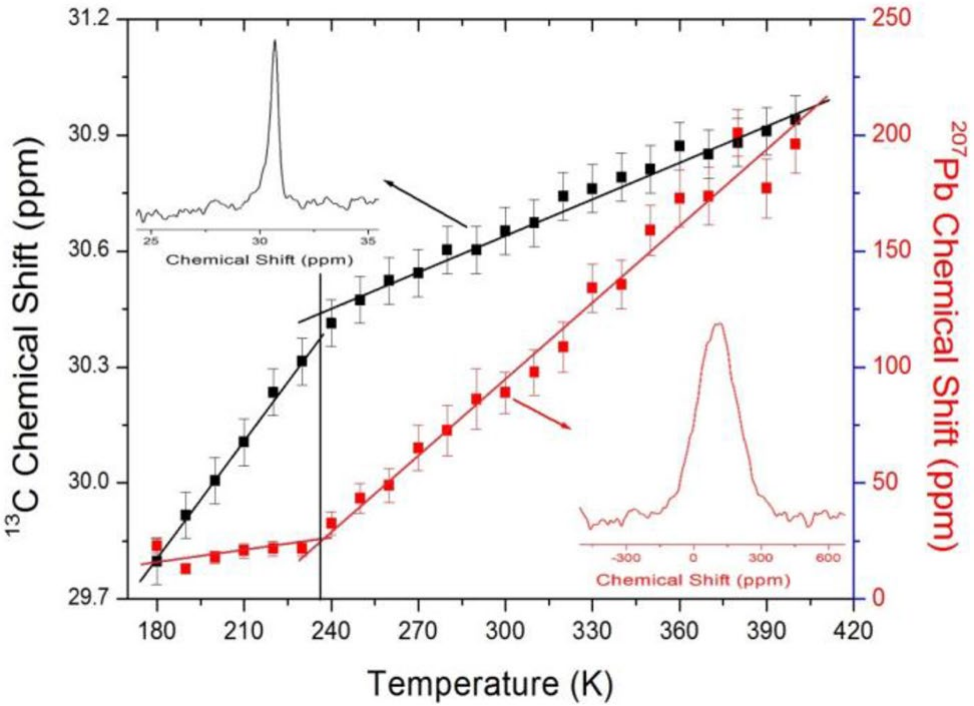
^13^C and ^207^Pb chemical shifts in CH_3_NH_3_PbBr_3_ as a function of temperature (inset: ^13^C and ^207^Pb MAS NMR spectrum of CH_3_NH_3_PbBr_3_ at 300 K).

Structural analysis of the ^13^C and ^207^Pb nuclei in CH_3_NH_3_PbBr_3_ was performed by MAS NMR, and the corresponding spectra at 300 K are shown as insets in Fig. 5. At room temperature, the ^13^C and ^207^Pb MAS NMR spectra show one signal each at chemical shifts of δ = 30.66 and 89 ppm with respect to tetramethylsilane and PbNO_3_, respectively. Here, the line width for ^13^C at 300 K is narrow at 2.77 ppm, whereas that for ^207^Pb is quite broad at 206.24 ppm. Figure 5 shows the ^13^C and ^207^Pb chemical shifts of CH_3_NH_3_PbBr_3_ measured as a function of temperature, illustrating that the ^13^C and ^207^Pb peak positions moved to higher chemical shifts upon heating. The chemical shifts near *T*_C_ changed, in contrast to the ^1^H chemical shifts. The chemical shifts of the ^13^C and ^207^Pb signals relative to the reference signal are sensitive to the electronic environment of the nucleus. In particular, the ^207^Pb chemical shift changed more rapidly than that of ^13^C near *T*_C_. From these results, the phase transition from the tetragonal to cubic phase is thought to arise from a change in the PbBr_6_ octahedra.

**Figure 6:**
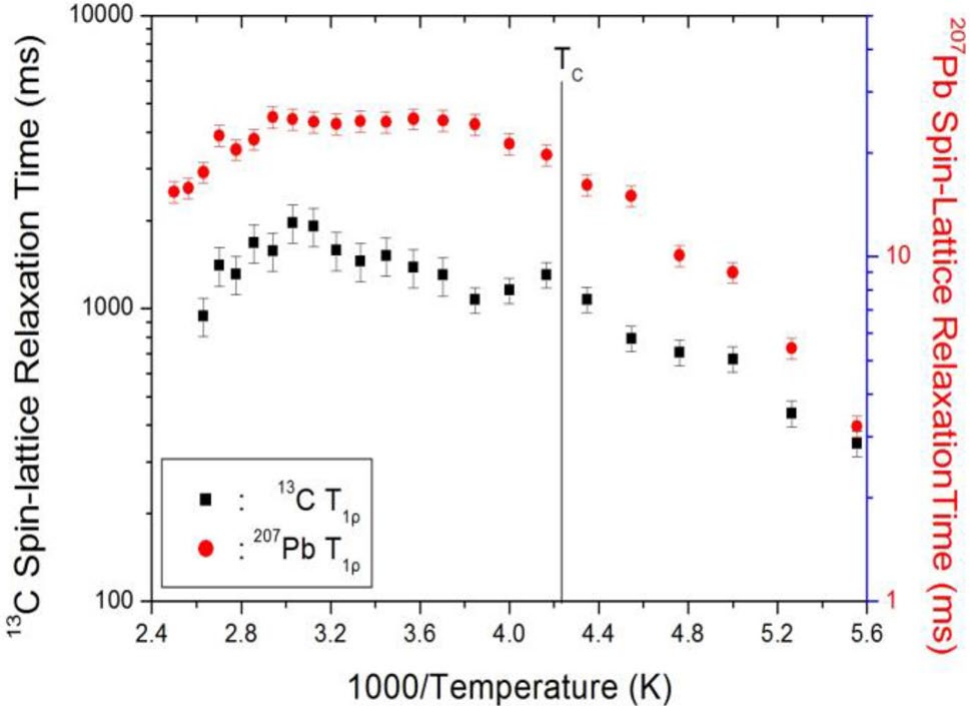
^13^C and ^207^Pb spin–lattice relaxation times T_1ρ_ of CH_3_NH_3_PbBr_3_ as a function of inverse temperature.

To determine the T_1ρ_ values of ^13^C and ^207^Pb in the rotating frame, the nuclear magnetisation was measured as a function of delay time. The signal intensities of the nuclear magnetisation recovery curves could be described by the single exponential function in Eq. (1), and the signal intensity followed this single exponential decay at all temperatures. From these results, the T_1ρ_ values were obtained for ^13^C and ^207^Pb in CH_3_NH_3_PbBr_3_ as a function of inverse temperature, as shown in Fig. 6. The ^13^C and ^207^Pb T_1ρ_ values for CH_3_ and PbBr_3_ seem to follow a similar trend with temperature to that of the ^1^H T_1ρ_, where the values increase with increasing temperature and are approximately continuous near *T*_C_. In addition, the ^207^Pb T_1ρ_ values are much lower than ^13^C T_1ρ_.

**Figure 7:**
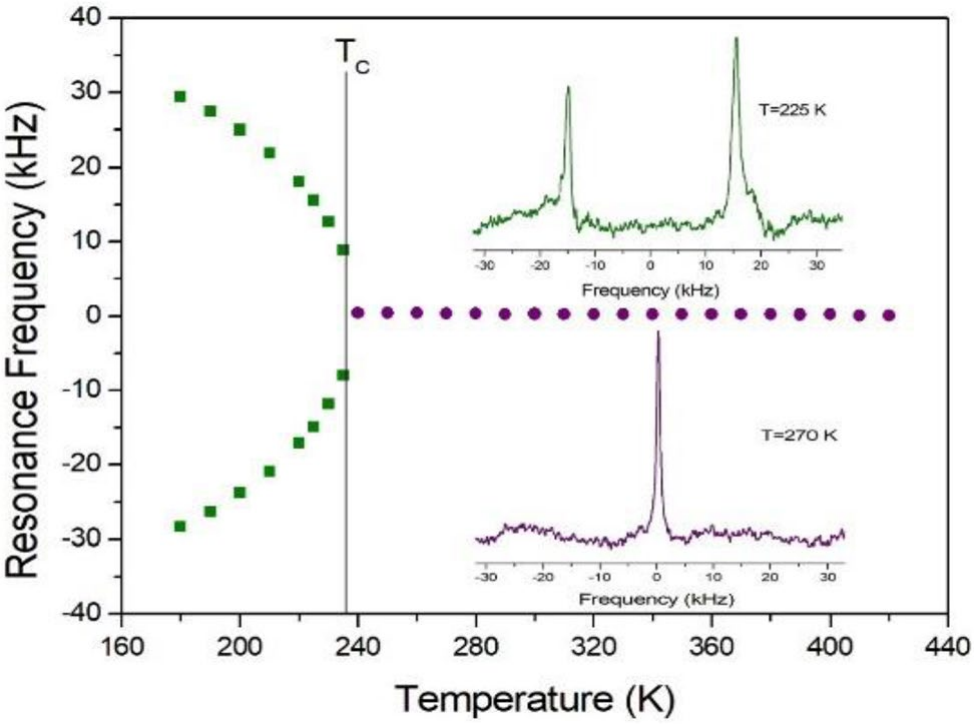
The ^14^N resonance frequency of CH_3_NH_3_PbBr_3_ single crystal as a function of temperature (inset: ^14^N NMR spectrum at tetragonal phase of 225 K and cubic phase of 270 K).

To obtain information concerning possible changes in the surroundings of the ^14^N ion, static NMR spectra of ^14^N (I = 1) in the laboratory frame were obtained. Temperature-dependent changes in the ^14^N resonance frequency are attributable to alterations in the structural geometry, indicating a change in the quadrupole coupling constant of the ^14^N nuclei. The spectra were obtained by the solid-state echo method using static NMR at a Larmor frequency of 28.90 MHz. Two resonance signals were expected from the quadrupole interactions of the ^14^N nucleus with spin I = 1. The ^14^N NMR spectra were shown at 225 and 270 K, and the resonance frequencies referenced with respect to NH_4_NO_3_ as a function of temperature are shown in Fig. 7. The line widths are very narrow at all temperatures. The two resonance signals for ^14^N, which are attributable to NH_3_, superpose into one line at the transition point of 236 K. This single ^14^N resonance line indicates that a phase transition takes place to a new phase with a higher symmetry than tetragonal^28^. In tetragonal phase below T_C_, the electric field gradient tensors at the N sites vary, reflecting changes in the atomic configuration around the nitrogen. But, there is no electric field gradient tensor at the ^14^N site in the cubic structure because of the site symmetry of m3m.

## Conclusion

Using the information derived from NMR studies near *T*_C_ (= 236 K), we have probed the structural and dynamic features of CH_3_NH_3_PbBr_3_ in detail and demonstrated its dynamic nature. The ionic dynamics of CH_3_NH_3_PbBr_3_, with emphasis on the role of the CH_3_NH_3_ cation and PbBr_6_ octahedra, were investigated by ^1^H MAS NMR, ^13^C CP/MAS NMR, ^207^Pb MAS NMR, and ^14^N static NMR as a function of temperature. ^1^H, ^13^C, and ^207^Pb NMR were used to identify the phase transition in CH_3_NH_3_PbBr_3_ by detecting changes in the chemical shifts accompanying a change in crystallographic symmetry. Here, the CH_3_ and NH_3_ groups were distinguished based on the ^1^H chemical shifts. The chemical shifts of the ^1^H nuclei remained constant at all temperatures, whereas those of the ^13^C and ^207^Pb nuclei varied with temperature. The temperature dependence of the chemical shifts was sensitive to the rotation of the PbBr_6_ octahedra. From these results, it is evident that the structural environments of ^13^C and ^207^Pb change near *T*_C_. The change in ^207^Pb chemical shift near *T*_C_ can be explained by the rotation of PbBr_3_. This is consistent with the established nature of the phase transition. Additionally, the NMR line widths of ^1^H, ^13^C, and ^207^Pb were 1.62, 2.77, and 206.24 ppm, respectively, and the relaxation time is proportional to the inverse of the line width. Although the chemical shifts of ^13^C and ^207^Pb abruptly varied near *T*_*C*_, the ^1^H, ^13^C, and ^207^Pb T_1ρ_ values showed a similar trend with increasing temperature, and their T_1ρ_ values were continuous near *T*_*C*_. These short relaxation times indicate ease of molecular motion. The TGA results also showed that CH_3_NH_3_PbBr_3_ has a high thermal stability.

In addition, the abrupt change occurring in the resonance frequency of the ^14^N nuclei near *T*_C_ is attributable to a structural phase transition. The NH_3_ groups in the structure are coordinated by PbBr_6_, and thus atomic displacements in the environment of the ^14^N nuclei with temperature are correlated with PbBr_6_. The electrostatic interactions governed by hydrogen-bonding interactions between the NH_3_^+^ group in the CH_3_NH_3_ cation and the PbBr_6_ octahedra play an important role in the dynamics of the CH_3_NH_3_ cations. Consequently, the main factor causing the phase transition from the tetragonal to cubic phase near *T*_C_ is a change in the surroundings of the ^207^Pb nuclei in the PbBr_6_ octahedra and in the surroundings of C–N groups in the CH_3_NH_3_ cations. Based on these results, the structural dynamics within the CH_3_NH_3_PbBr_3_ perovskite structure are expected to have a significant effect on the operation mechanism of perovskite solar cells.

## Supplementary information

Supplementary Information.
