# Supplementary material for: Structural dynamics of CH3NH3+ and PbBr3− in tetragonal and cubic phases of CH3NH3PbBr3 hybrid perovskite by nuclear magnetic resonance
Source: Sci Rep. 2020 Aug 4;10:13140. doi: 10.1038/s41598-020-70128-5 (PMC7403148; doi:10.1038/s41598-020-70128-5)
Supplement: Supplementary file 1 — Supplementary Information. [file 41598_2020_70128_MOESM1_ESM.docx]

Structural dynamics of CH_3_NH_3_^+^ and PbBr_3_^-^ in tetragonal and cubic phases of CH_3_NH_3_PbBr_3_ hybrid perovskite by nuclear magnetic resonance

Ae Ran Lim^*a^ Sun Ha Kim,^b,c^ Yong Lak Joo^d^

^a^ Analytical Laboratory of Advanced Ferroelectric Crystals, and Department of Science Education, Jeonju University, Jeonju 55069, Korea. Corresponding author (E-mail: aeranlim@ hanmail.net, [arlim@jj.ac.kr)](mailto:arlim@jj.ac.kr)), Tel.: +82-(0)63-220-2514

^b^ Seoul Western Center, Korea Basic Science Institute, Seoul 03759, Korea

^c^ Department of Chemistry, Kyungpook National University, Daegu 41566, Korea

^d^ Robert Fredrick Smith School of Chemical and Biomolecular Engineering, Cornell University, Ithaca, New York 14853, USA


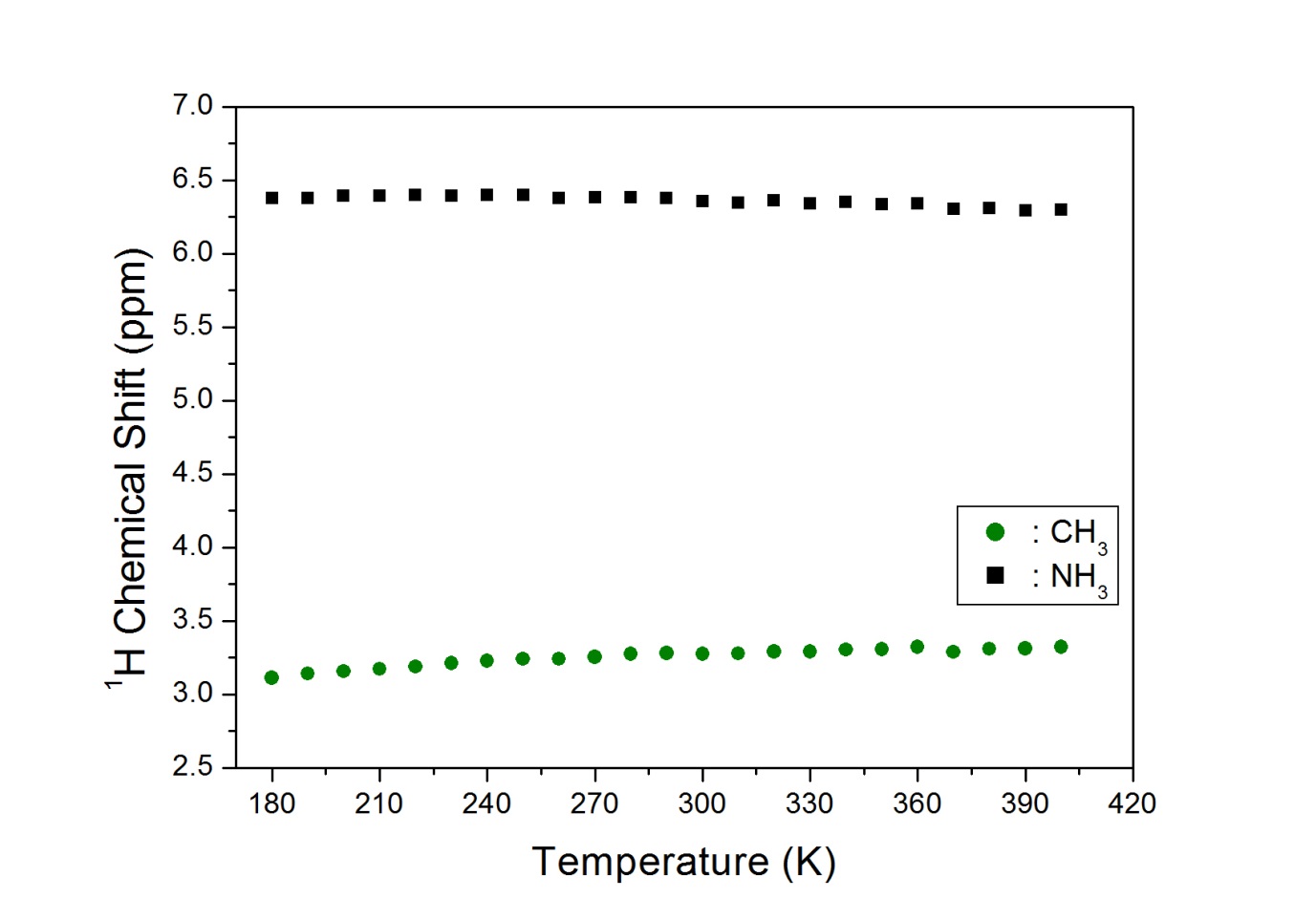


Figure. ^1^H chemical shifts for CH_3_ and NH_3_ in CH_3_NH_3_PbBr_3_ as a function of temperature.
